#  Relationship between super antigenicity, antimicrobial resistance and origin of Staphylococcus aureus isolated 

**Published:** 2016-03-30

**Authors:** Luisa Fernanda Corredor Arias, Jenna Samara Luligo Espinal, José Ignacio Moncayo Ortiz, Jorge Javier Santacruz Ibarra, Adalucy Álvarez Aldana

**Affiliations:** 1 Montana State University. Montana USA; 2 Centro de Biología Molecular y Biotecnología. Facultad de Ciencias de la Salud Universidad Tecnológica de Pereira, Pereira Colombia; 3 Facultad de Ciencias de la Salud. Universidad Tecnológica de Pereira, Pereira Colombia

**Keywords:** Staphylococcus aureus, hospital infection, toxin, antibiotic resistance, microbial sensivity, genotype, genetic variation

## Abstract

**Introduction:**

*Staphylococcus aureus* is a pathogen that causes food poisoning as well as hospital and community acquired infections.

**Objective:**

Establish the profile of superantigen genes among hospital isolates in relation to clinical specimen type, susceptibility to antibiotics and hospital or community acquisition.

**Methods:**

Eighty one isolates obtained from patients at Colombian hospital, were classified by antimicrobial susceptibility, specimen type and hospital or community acquired . The PCR uniplex and multiplex was used for detection of 22 superantigen genes (18 enterotoxins, *tsst-1* and three exfoliative toxins).

**Results:**

Ninety five point one percent of isolates harbored one or more of the genes with an average of 5.6 genes. Prevalence of individual genes was variable and the most prevalent was *seg* (51.9%). Thirty nine genotypes were obtained, and the genotype *gimnou* (complete *egc *cluster) was the most prevalent alone (16.0%) and in association with other genes (13.6%). The correlation between presence of superantigens and clinical specimen or antimicrobial susceptibility showed no significant difference. But there was significant difference between presence of superantigens and the origin of the isolates, hospital or community acquired (*p*= 0.049).

**Conclusions:**

The results show the variability of the superantigen genes profile in hospital isolates and shows no conclusive relationship with the clinical sample type and antimicrobial susceptibility, but there was correlation with community and hospital isolates. The analysis of the interplay between virulence, epidemic and antibiotic resistance of bacterial populations is needed to predict the future of infectious diseases.

## Abstract (es)

**Introducción:**

*Staphylococcus aureus,* es un patógeno que causa intoxicación alimentaria e infecciones hospitalarias y comunitarias.

**Objetivo:**

Establecer el perfil de genes de superantígenos en aislamientos hospitalarios correlacionándolos con el tipo de muestra clínica, susceptibilidad antimicrobiana y origen hospitalario o comunitario.

**Métodos:**

Se analizaron 81 aislamientos de *S. aureus* de pacientes de un hospital colombiano. Fueron clasificadas por susceptibilidad antimicrobiana, tipo de muestra clínica y origen hospitalario o comunitario. Se detectó por PCR individual y múltiple 22 genes de superantígenos (18 enterotoxinas, una toxina del choque tóxico-1 y tres toxinas exfoliativas).

**Resultados:**

El 95.1% albergaban uno o más genes de superantígenos con un promedio de 5.6 genes. La prevalencia individual fue variable y el gen con mayor prevalencia fue *seg *(51.9%). Se obtuvieron 39 genotipos, y el genotipo *gimnou *(*cluster egc *completo) fue el de mayor frecuencia (16.0%) y asociado con otros genes (13.6%). La correlación de superantígenos frente a tipo de muestra clínica y susceptibilidad antimicrobiana no mostró diferencia estadística significativa, pero hubo diferencia significativa con el tipo de aislamiento hospitalario o comunitario (*p*= 0.049).

**Conclusiones:**

Los resultados muestran la diversidad genética en los aislados hospitalarios respecto a la presencia de superantígenos y no muestra una relación concluyente con el tipo de muestra clínica y susceptibilidad antimicrobiana pero sí con origen de los aislamientos comunitarios y hospitalarios. Un análisis de la interrelación entre la virulencia, epidemicidad y resistencia antimicrobiana de las poblaciones bacterianas es necesario para predecir el futuro de las enfermedades infecciosas.

## Introduction

*Staphylococcus aureus* is a major human pathogen capable of causing a wide range of infections, including skin and soft tissue infections, Healthcare-associated infections (nosocomial infections), food poisoning and life-threatening infections such as toxic shock syndrome, endocarditis, osteomyelitis, meningitis and pneumonia 1,2.

The pathogenicity of *S. aureus* is very complex, involving numerous bacterial products and sophisticated regulatory pathways 3. Many virulence factors have been described, such as antibiotic resistance, production of exotoxins and enzymes that contribute to its ability to colonize and cause disease 1,4. 

Some strains produce one or more exoproteins, including staphylococcal enterotoxins (SE), toxic shock syndrome toxin (TSST-1), exfoliative toxins (ET) and leukocidins 4. 

Superantigenicity is one of the most studied properties of this exoproteins, which refers to its ability to activate 20 to 30% of T lymphocytes with a massive production of pro-inflammatory cytokines and chemokines that can cause fever, hypotension and other disorders including potentially lethal shock 1,5,6.

There are five known types of classical enterotoxins (SEA to SEE), many new types of enterotoxins have been described, called nonclassical enterotoxins (SEG to SEU) 1,4,7-10 and more recently described, superantigen SElX (*selx gene*), that contributes to the lethality of a strain CA-MRSA in necrotizing pneumonia (community acquired-Methicillin Resistant *S. aureus*) 11.

Most types of enterotoxins are encoded in mobile genetic elements located on pathogenicity islands, transposons, plasmids, phages or highly variable genetic regions (vSaβ) 6,11. There is an enterotoxin gene cluster (*egc*) that groups the genes *seg-sei-sem-seo-sen* and sometimes *seu*, which has been linked to increased virulence of *S. aureus *strains and is the most commonly detected. It is located in the pathogenicity island vSA 9,12,13. Other genes of enterotoxins are detected in the methicillin resistance chromosomal cassette (*SCCmec*) 14.

Exfoliative toxins produced by *S. aureus* are responsible for the scalded skin syndrome (SSS), which causes the most sever bullous skin manifestations. It primarily affects infants and young children 15,16. ETs are classified as ETA, ETB, and ETD, ETA and ETB. ETB being the most frequent 17,18 and ETD the less frequent 19.

Recent studies show the prevalence of superantigen genes in *S. aureus* isolates from different sources and geographical regions. Some clinical studies have attempted to correlate the results of detection of superantigen genes in patients with *S. aureus* infections, but their relationship has not been elucidated 5,20,21. 

The objective of the study was to establish the relationship between the profile of superantigen genes (SEs, ETs and TSST-1) to antiobiotic resistance, to clinical specimen type (blood, secretions, others specimens), and to hospital (HA: Hospital-Acquired) or community (CA: Community-Acquired) in isolates of *S. aureus* in order to try to establish the potential virulence of hospital isolates.

## Materials and Methods

A prospective study was carried out with 81 isolates obtained between February 2009 and March 2011. Only *S. aureus* isolates from patients with diagnosis of Healthcare-associated infections of both sex and adult were included and excluded patients with polymicrobial infections and children. We analyzed only the first isolation cultured by the clinical laboratory. The study was approved and supervised by the Bioethics Committee of the Faculty of Health Sciences - Technological University of Pereira.

The identification of clinical isolates and antibiotic susceptibility were performed by Automated Systems for Susceptibility Testing (WalkAway/Microscan^®^, Dade Behring) at clinical laboratory of the Hospital Universitario San Jorge (Pereira-Colombia), this hospital is an institution providing health services II, III and IV levels of care, with 402 hospital beds and an average monthly hospital admissions and discharge of 2,100 and 1,700 patients, respectively. The levels of care correspond to the therapies and services provided.

The isolates were confirmed as *S. aureus* by PCR using primers: Sa442-1 5'-AAT CTT TGT CGG TAC ACG ATA TTC TTC ACG -3' and Sa442-2 5'-CGT AAT GAG ATT TCA GTA GAT AAT ACA ACA -3', to amplify a portion of the species-specific gene Sa442 of *S. aureus* 22. 

### Classification of S. aureus isolates

The isolates were classified according to the type of clinical specimen (blood, secretions, and others). Secretions which come from ears, eyes, muscle, burns, scalp, joint regions, osteosynthesis material, abscesses and wounds. Samples of devices such as catheters and tracheobronchial aspirates, pleural, peritoneal and cerebrospinal fluid and urine were classified as others. The isolates were also classified as resistance to one or more antibiotics and finally isolates were classified by the Hospital Universitario San Jorge as home hospital (HA) or community (CA).

### DNA extraction

The CTAB (cetyltrimethylammonium bromide) genomic DNA extraction method was modified to use lysostaphin and the methodology described by Johnson *et al* 23. The extracted DNA was stored at -20^°^ C in aliquots of 20 ng/μL for subsequent analysis by PCR

### Identification of superantigen genes

All isolates were examined for 22 genes (*sea to see, tsst-1, seg to ser, seu, eta, etb and etd)* by PCR. Five reference strains were used as positive controls, containing one or more superantigens genes: ATCC 700699, ATCC BAA-1707, ATCC 13565, ATCC 13566 and ATCC 19095 (FRI137).

The nucleotide sequences for all the primers used in this study and their respective amplification products are described for other authors 10,18,24 **(** Table 1).

**Table 1.** Primers, nucleotide sequence and the size of PCR amplification products.

Gene	Primer sequence (5´- 3´)	Size (bp)	Reference
*sea*	GGT TAT CAA TGT GCG GGT GG	102	24
	CGG CAC TTT TTT CTC TTC GG		
*seb*	GTA TGG TGG TGT AAC TGA GC	164	24
	CCA AAT AGT GAC GAG TTA GG		
*sec*	AGA TGA AGT AGT TGA TGT GTA TGG	451	24
	CAC ACT TTT AGA ATC AAC CG		
*sed*	CCA ATA ATA GGA GAA AAT AAA AG	278	24
	ATT GGT ATT TTT TTT CGT TC		
*see*	AGG TTT TTT CAC AGG TCA TCC	209	24
	CTT TTT TTT CTT CGG TCA ATC		
*tst*	ACC CCT GTT CCC TTA TCA TC	326	24
	TTT TCA GTA TTT GTA ACG CC		
*eta*	GCA GGT GTT GAT TTA GCA TT	93	24
	AGA TGT CCC TAT TTT TGC TG		
*etb*	ACA AGC AAA AGA ATA CAG CG	226	24
	GTT TTT GGC TGC TTC TCT TG		
*etd*	AAC TAT CAT GTA TCA AGG	376	18
	CAG AAT TTC CCG ACT CAG		
*seg*	AAGTAGACATTTTTGGCGTTCC	287	25
	AGAACCATCAAACTCGTATAGC		
*seh*	GTCTATATGGAGGTACAACACT	213	25
	GACCTTTACTTATTTCGCTGTC		
*sei*	GGTGATATTGGTGTAGGTAAC	454	25
	ATCCATATTCTTTGCCTTTACCAG		
*sej*	ATAGCATCAGAACTGTTGTTCCG	152	25
	CTTTCTGAATTTTACCACCAAAGG		
*sek*	TAGGTGTCTCTAATAATGCCA	293	25
	TAGATATTCGTTAGTAGCTG		
*sel*	TAACGGCGATGTAGGTCCAGG	383	25
	CATCTATTTCTTGTGCGGTAAC		
*sem*	GGATAATTCGACAGTAACAG	379	25
	TCCTGCATTAAATCCAGAAC		
*sen*	TATGTTAATGCTGAAGTAGAC	282	25
	ATTTCCAAAATACAGTCCATA		
*seo*	TGTGTAAGAAGTCAAGTGTAG	214	25
	TCTTTAGAAATCGCTGATGA		
*sep*	TGATTTATTAGTAGACCTTGG	396	25
	ATAACCAACCGAATCACCAG		
*seq*	AATCTCTGGGTCAATGGTAAGC	122	25
	TTGTATTCGTTTTGTAGGTATTTTCG		
*ser*	GGATAAAGCGGTAATAGCAG	166	25
	GTATTCCAAACACATCTAAC		
*seu*	ATTTGCTTTTATCTTCAT	167	10
	GGACTTTAATGTTTGTTTCTGAT		
*fem A*	AAAAAAGCACATAACAAGCG	132	24
	GATAAAGAAGAAACCAGCAG		
*fem B*	TTACAGAGTTAACTGTTACC	651	25
	ATACAAATCCAGCACGCTCT		

### Multiplex PCR

Two sets of multiplex PCR were used to detect classical enterotoxins genes (*sea to see*), toxic shock syndrome toxin (*tsst-1*) and exfoliative toxins (*eta y etb*). The internal control of the reactions was a couple of primers femA, that amplified a 132 bp fragment of the *femA* gene (structural component of peptidoglycan, associated to methicillin resistance) 10,18,24. Reference ATCC strains were used as positive controls and sterile distilled water as negative control.

Multiplex PCR series A included primers for genes *sea, seb, sec, sed, see and femA.* The amplification reaction was performed in a final volume of 25 µL, containing 1X buffer (50 mM KCl, 10 mM Tris-HCl, pH 9.0), 3 mM MgCl_2_, 200 µM dNTPs, 20 pmol each of the primers *sea, seb, sec and see,* 40 pmoles of primer *sed*, 1 U of Taq polymerase (Invitrogen) and 1 µL of DNA at a concentration of 20 ng/µL. Amplification was performed in a thermocycler (Perkin Elmer GeneAmp 9700), under the following conditions: denaturation at 94^º^ C for 1 min, annealing at 61^º^ C for 30 s and extension at 74^º^ C for 1 min, for 35 cycles 24.

Multiplex PCR series B included the primers for exfoliative toxins A and B genes and the toxic shock syndrome toxin (TSST-1). The amplification reaction had the same constituents and concentrations of the amplification reaction for the series A, except for the primer *eta* that must be 50 pmoles, as stated in Mehrotra *et al* 24. For *tst-1* and *etb* is 20 pmoles. Amplification temperatures and cycles are as described for the set A.

### Individual PCR (Uniplex)

Was used to detect nonclassical enterotoxin genes: *seg to ser, seu, *and exfoliative toxin *etd*. Gene *FemB* primers were used as an internal control of the reactions, which is also an essential factor for the expression of methicillin resistance 25. Reference ATCC strains were used as positive control and sterile distilled water negative control.

The reaction mixture (25 µL) consisted of 20 pmol of each primer, 2.5 mM MgCl_2_, 200 µM of each oligonucleotide, 1 U of Taq DNA polymerase and 1X buffer. Thermocycler conditions were: denaturation at 94^º^ C for 30 s, annealing at 55^º^ C for 30 s, and extension at 72^º^ C for 60 s for 30 cycles 25.

Detection and analysis of all the amplified products was performed by 2% agarose gel electrophoresis stained with ethidium bromide.

### Statistical analysis

Contingency tables were prepared and different correlations were analyzed between the presence or absence of superantigens genes against antimicrobial resistance, clinical specimen type, source of isolation of *S. aureus, *hospital or community acquired. Fisher's exact test was applied and *p *≤0.05 with a confidence interval of 95%, were considered statistically significant. 

##  Results

Eighty one isolates of *S. aureus *identified by Automated Systems for Susceptibility Testing and were confirmed by Sa442 gene amplification (100%). The distributions of isolates between genders were 48.1% (39/81) for male with an average of 6.2 genes detected and 51.9% (42/81) for female with an average of 4.9 genes. No statistically significant difference (*p*= 0.37).

### Classification of S. aureus isolates according to the type clinical specimen

Clinical isolates of *S. aureus* were classified according to the type of clinical sample: 23 blood isolates (28.4%), 39 from secretions (48.1%) and 19 clinical specimens were classified as others (23.5%). 

### Antimicrobial susceptibility in S. aureus isolates

Antimicrobial susceptibility indicated that 6 isolates (7.4%) were susceptible to all 12 antimicrobial group´s (26 antimicrobials) tested and 75 (92.6%) were resistant to one or more antibiotics. The resistance range varied from 92.6% (75/81) for Beta- Lactam to 1.2% (1/81) to rifamycin. Additionally, 31 isolates was resistant for oxacillin (38.7%) phenotypically classified as MRSA isolates (Table 2).

**Table 2.** Antimicrobial group's susceptibility in *S. aureus* isolates. N= 81

Antimicrobial group	S	%	R	%
Beta-Lactam	6	7.4	75.0	92.6
Chloranphenicol	81	100	0	0.0
Quinolones	54	66.7	277	33.3
Clindamycin	54	66.7	277	33.3
Macrolide	43	53.1	381	46.9
Fusidic acid	81	100	0	0.0
Aminoglycoside	56	69.1	251	30.9
Nitrofurantoin	81	100	0	0.0
Rifamycin	80	98.8	1	1.2
Tetracycline	59	72.8	228	27.2
Trimethoprim/Sulfonamide	81	100	0	0.0
Glycopeptide	81	100	0	0.0

S= sensivity

R= resistance

### Prevalence and distribution of the genes encoding superantigens

The prevalence of the 22 genes detected by PCR in the 81 isolates of *S. aureus *was 95.1% (77/81) and in 4.9% (4/81) no genes were detected. The range of genes detected was at least 2 genes in 11 isolates (13.6%) and up to 13 genes in 6 isolates (7.5%), for an average of 5.9 genes.

The distribution of genes showed 39 genotypes and the genotype *gimnou* (complete *egc *cluster) was the most prevalent (16.0%) and associated with other genes (13.6%) followed by *sek-seq* with 18.4% and 42.0% of the genotypes had different combinations of genes.

The prevalence of individual genes was variable and *seg* was the most prevalent gene (51.9%), followed by *seq* (45.7%) and the lowest prevalence was *eta* (2.5%). No *sep, ser *or *etb* genes were detected. The difference was marked between classical enterotoxins with a gene average of 0.44 and with 4.0 for nonclassical genes (Table 3).

**Table 3.** Profile of superantigen genes distributed as classical, nonclassical, toxic shock syndrome toxin and exfoliative toxins in clinical isolates of *S. aureus*.

Variables	Gene	NP	%
Classical superantigens	sea	9	11.1
	seb	8	9.9
	sec	13	16.0
	sed	3	3.7
	see	3	3.7
Toxic shock syndrome toxin	tsst-1	5	6.2
Nonclassical superantigens	seg	42	51.9
	seh	29	35.8
	sei	36	44.4
	sej	3	3.7
	sek	25	30.9
	sel	18	22.2
	sem	27	33.3
	sen	36	44.4
	seo	30	37.0
	seq	37	45.7
	seu	41	50.6
Exfoliative toxin	eta	2	2.5
	etd	34	42.0

NP= number positive

### Correlation between the presence of superantigen genes and antimicrobial susceptibility

In 6 antimicrobial susceptible isolates 6.2% (5/81) superantigen genes were detected and 1.2% had none. In resistant, 92.6% had them, in the latter case there were no resistant isolates without superantigens. No significant difference (*p*= 0.074) (Table 4).

**Table 4.** Relationship between superantigens genes with antimicrobial susceptibility, hospital or community origin and type of clinical sample.

Superantigens	Antimicrobial susceptibility				Acquired place				Type clinical specimen					
	S	%	R	%	HA	%	CA	%	Blood	%	Secretion	%	Other	%
SAg+	5	6.2	75	92.6	35	43.2	42	51.9	21	25.9	37	45.7	19	23.5
SAg-	1	1.2	0	0.0	4	4.9	0	0.0	2	2.5	2	2.5	0	0.0
Total	6	7.4	75	92.6	39	48.1	42	51.9	23	28.4	39	48.1	19	23.5
p				0.074				0.049						0.566

SAg: superantigen. S: susceptible; R: resistantance. HA: Hospital-Acquired; CA: Community-Acquired. *p*: Fisher's exact test.

Beta-Lactam were the only group that showed statistically significant difference (*p* <0.05) in the correlation between the superantigens genes and antimicrobial group's susceptibility in* S. aureus *isolates.

### Correlation between the presence of superantigen genes and type of hospital and community isolate

Table 4 shows the detection of superantigen genes according to the origin HA or CA, where 43.2% (35/81) isolates for HA had one or more SAg genes and 59.9% (42/81) of CA isolates had one or more superantigens genes of with statistically significant difference (*p*= 0.049).

### Correlation between the presence of superantigen genes and the type of clinical sample

Of the 23 isolates from blood specimens, 21 (25.9%) had one or more genes with an average of 5.2 superantigen genes. For secretion, of the 39 isolates, 37 (45.7%) genes were detected with an average of 5.8 genes, and for the 19 isolates classified as other specimens, all genes were detected (23.5%) with an average of 6.9 genes. Statistical analysis by Fisher's exact test showed no significant difference (*p*= 0.566) (Table 4).

## Discussion

*Staphylococcus aureus* is the second most isolated pathogen from infections at the Hospital Universitario San Jorge de Pereira as well as in all the hospitals around Colombia 26. 95.1% of all isolates contained at least one superantigen gene. High prevalence has also been reported by Varshney *et al*., 99% 5, Chiang *et al*., 91.8% 10, Xie *et al*., 90.7% 27 and Omoe *et al*., 77.4% 25. 

The prevalence of individual genes varied as reported by other researchers 5,6,10. The most common single gene in the study was* seg* (51.9%) which also has been reported by other studies 6,10, 25. In Colombia, Portillo *et al.* 28, found the *seg *gene at 94% in isolated MRSA.

For other genes, in contrast to Portillo, we detected *tsst, sea, seb, sec, seh* and *sel *but not *sep, ser *and* etb* genes, this indicates heterogeneity genetic of *S. aureus* intro the same country. This indicates that the presence of individual genes or associations between them is very diverse in all isolates of *S. aureus* regardless of origin.

The number of genotypes detected was 39, similar to that reported by Omoe *et al*. 25, and greater than that reported by Kuroda *et al*. 9, which allows us to infer that the number of genotypes is variable and dependent to the type of strain and the geographic area from which the isolate proceeded. 

The presence of the full *egc* cluster or in coexistence with other SAgs in this study is consistent with other reports of its prevalence 5,6,12,29 and lower than that reported by Portillo *et al*. 28, An interesting fact was the detection of the gene couple *sek-seq* (18.5%) associated with phage φ-3 followed in frequency the *egc* cluster; the couple *sed-sej* encoded on plasmid pIB485 reported by Varshney *et al*., was not found 5. 

In this study no significant differences were established between the presence of superantigens and type of clinical specimen (blood, secretions, and others) and the same happened when compared with antimicrobial susceptibility. The only relationship that showed a significant difference was the comparison between the presence of superantigens against hospital (HA) or community (CA) origin.

## Conclusions

The high detection and the variety of arrangements of superantigen genes in hospital isolates of *S. aureus* demonstrate their virulence, and their presence allows differentiation between CA and HA isolates. 

No correlation was established between superantigens and carry genes for antibiotic resistance or type of clinical specimen. In future studies it will be necessary to establish the importance of the *egc *cluster as a possible marker for virulence in *S. aureus* isolates. 

It is important to note that one limitation of the study was unable to establish superantigen gene expression in vitro. The detection de superantigens proteins will be made in future research to determine the true virulence in hospital isolates of *S. aureus*. 

Today, the analysis of the interplay between virulence, epidemic and antibiotic resistance of bacterial populations is needed to predict the future of infectious diseases.
